# Psychological morbidity, sources of stress and coping strategies among undergraduate medical students of Nepal

**DOI:** 10.1186/1472-6920-7-26

**Published:** 2007-08-02

**Authors:** Chandrashekhar T Sreeramareddy, Pathiyil R Shankar, VS Binu, Chiranjoy Mukhopadhyay, Biswabina Ray, Ritesh G Menezes

**Affiliations:** 1 Department of Community Medicine, Manipal College of Medical Sciences, Pokhara, Nepal; 2 Department of Pharmacology, Manipal College of Medical Sciences, Pokhara, Nepal; 3 Department of Community Medicine, Manipal College of Medical Sciences, Pokhara, Nepal; 4 Department of Microbiology, Kasturba Medical College, Manipal, India; 5 Department of Anatomy, Kasturba Medical College, Manipal, India; 6 Department of Forensic Medicine, Manipal College of Medical Sciences, Pokhara, Nepal

## Abstract

**Background:**

In recent years there has been a growing appreciation of the issues of quality of life and stresses involved medical training as this may affect their learning and academic performance. However, such studies are lacking in medical schools of Nepal. Therefore, we carried out this study to assess the prevalence of psychological morbidity, sources and severity of stress and coping strategies among medical students in our integrated problem-stimulated undergraduate medical curriculum.

**Methods:**

A cross-sectional, questionnaire-based survey was carried out among the undergraduate medical students of Manipal College of Medical Sciences, Pokhara, Nepal during the time period August, 2005 to December, 2006. The psychological morbidity was assessed using General Health Questionnaire. A 24-item questionnaire was used to assess sources of stress and their severity. Coping strategies adopted was assessed using brief COPE inventory.

**Results:**

The overall response rate was 75.8% (407 out of 525 students). The overall prevalence of psychological morbidity was 20.9% and was higher among students of basic sciences, Indian nationality and whose parents were medical doctors. By logistic regression analysis, GHQ-caseness was associated with occurrence of academic and health-related stressors. The most common sources of stress were related to academic and psychosocial concerns. The most important and severe sources of stress were staying in hostel, high parental expectations, vastness of syllabus, tests/exams, lack of time and facilities for entertainment. The students generally used active coping strategies and alcohol/drug was a least used coping strategy. The coping strategies commonly used by students in our institution were positive reframing, planning, acceptance, active coping, self-distraction and emotional support. The coping strategies showed variation by GHQ-caseness, year of study, gender and parents' occupation.

**Conclusion:**

The higher level of psychological morbidity warrants need for interventions like social and psychological support to improve the quality of life for these medical students. Student advisors and counselors may train students about stress management. There is also need to bring about academic changes in quality of teaching and evaluation system. A prospective study is necessary to study the association of psychological morbidity with demographic variables, sources of stress and coping strategies.

## Background

Students are subjected to different kinds of stressors, such as the pressure of academics with an obligation to succeed, an uncertain future and difficulties of integrating into the system. The students also face social, emotional and physical and family problems which may affect their learning ability and academic performance [1,2]. Too much stress can cause physical and mental health problems, reduce students self-esteem and may affect students academic achievement [3,4]. In recent years there is a growing appreciation of the stresses involved in medical training. Studies have classified the sources of stress into three main areas: academic pressures, social issues and financial problems [5]. In addition to educating in a professional medical course it is also important to take into account the quality of life of the students during the years of medical training. Earlier studies have emphasized this point [5-7].

Studies from United Kingdom, that have examined coping strategies of medical students with the stresses of undergraduate education have generally identified use of alcohol as a coping strategy [6-9] but some studies have reported the use of other substances such as tobacco and drugs [10,11]. But a study from Pakistan reported that sports, music and hanging out with friends were common coping strategies [12]. Stewart et al used the COPE, a multidimensional coping inventory which includes assessment of both problem-focused and emotion-focused coping strategies, in studies of Hong Kong Chinese medical students [13-15]. Studies from developing countries like Pakistan, India, Thailand and Malaysia have reported stress among medical students and have underscored the role of academics as a source of stress [12,16-18]. But these studies have either not assessed the coping strategies or did not use COPE inventory. A study from the United Kingdom has reported a higher rate of psychological morbidity and stressors related to medical training among the first year students in a new problem-based medical curriculum [19]. The study had used brief COPE to assess the coping strategies of medical students during the stressful events.

At the Manipal College of Medical Sciences (MCOMS), Pokhara, Nepal, there are students from Nepal, India, Sri Lanka and other countries. These students come from diverse cultural, socioeconomic and educational backgrounds. More than half the medical students are from other countries and they are exposed to a new learning environment, making new friends, and generally adapting to a new and somewhat uncertain world during their training at the medical school. This may be a stressful experience. Information on sources and severity of stress and coping strategies are lacking among medical students in medical schools. This information may aid in designing appropriate intervention strategies and planning modifications in the medical curricula to enhance the students' learning abilities. Hence the present study was undertaken with the following objectives:

1. Estimate the prevalence of psychological morbidity and

2. Identify the sources of stress, their severity and coping strategies.

## Methods

### Setting and participants

The present study was undertaken at MCOMS, affiliated to Kathmandu University. The seven basic science subjects (anatomy, physiology, biochemistry, pathology, microbiology, pharmacology and community medicine) are taught during the first two years of the MBBS (Bachelor of Medicine and Bachelor of Surgery) course in an integrated, organ system based manner. The university recommends a reduction in factual content and lecture-based teaching of medical course following global trends. The curriculum emphasizes problem-stimulated learning [20]. Every year MCOMS admits two batches of 75 students each for MBBS course in February and August. The students of February and August batches of the years 2004 and 2005 were invited to participate in the study. The authors look at reasons for refusal to participate in the study. A few possible reasons which emerged on discussion with certain students who did not participate were the sensitive and personal nature of the study, the length of the questionnaire and the students being out of station.

### Study design

A cross-sectional survey using an anonymous self-administered questionnaire. The institutional ethics committee of MCOMS approved this study. The study was carried out during August-December, 2005 among basic science students and during august-december, 2006 among clinical sciences students. Each batch of students was briefed about the purpose and objective of the study. Verbal consent was sought to participate in the study. The students were assured about anonymity and confidentiality of the responses given in the questionnaire and instructed to return the completed questionnaire. The questionnaire comprised of demographic data, the 12-item General Health Questionnaire (GHQ-12) [21], a 29-item list of potential stressors, and the Brief COPE [22] to determine coping strategies.

### Definitions of variables

#### GHQ Case

We followed standard method of defining the GHQ case. In the standard method individuals scored 0 if choosing either of the first two categories or 1 for choosing either the third or fourth category, with the scaled scores summed to produce a total score of 12. The threshold scores are set to correspond to a case definition equivalent to that of the average patient referred to a psychiatrist. Scores above the threshold are probable cases. For the present study we considered a threshold score of 4–5 as recommended by the authors [21].

#### Stressors

The potential stressors included in the questionnaire were derived by reviewing the literature and by holding informal discussion with a group of students. A total of 29 sources of stress were listed and grouped as academic, psychosocial and health-related. For each potential stressor, the frequency of occurrence was classified as never, rarely, sometimes, often and always and scored as 1, 2, 3, 4 and 5 respectively. The severity of each stressor was rated using a Likert scale (1–10) ranging from not severe to very severe.

#### Brief COPE

The Brief COPE responses ranging from "I have not been doing this at all" to "I have been doing this a lot" were scored from 1 to 4. The 24-item scores were averaged in pairs to produce 12 coping strategy scores [22]. Each pair of the coping strategy was used in the logistic regression analysis with GHQ-caseness and the stressor groups.

### Data analysis

Data were analyzed using SPSS for Windows (Version 10.0). The number and percentage of GHQ-12 cases were estimated according to demographic variables. Percentage frequency of occurrence was calculated for each of the stressors. Descriptive statistics were calculated for severity of sources of stress and coping strategies. Univariate and logistic regression analysis was carried out to determine the predictors of GHQ-caseness. A stepwise logistic regression analysis was carried out by comparing GHQ-caseness and occurrence of each group of sources of stress with scores of 12 pairs of coping strategies.

## Results

### Basic demographics

A total of 525 students (i.e. 290 basic science students and 235 clinical science students) were invited to complete the questionnaires. A total of 407 students (i.e. 239 basic science and 168 clinical science students) completed and returned the questionnaires. Four questionnaires were not considered for analysis since demographic data and other important responses in the questionnaires were incomplete. The overall response rate was 75.8% (basic science 82.4% and clinical science 71.5%). The mean age of the respondents was 20.7 years (SD = 1.8) with a range of 17–29 years. Two hundred and twenty seven (56.3%) students were males and 176 (43.7%) were females. Among the nationalities, 198 (49.1%) were Indians, 164 (40.7%) were Nepalese and 41 (10.2%) were from Sri Lankan and other countries.

### Psychological morbidity

The overall prevalence of psychological morbidity was 20.9%. The difference in proportion of GHQ-cases according to gender, year of study, basic science or clinical science was not statistically significant. By univariate analysis the difference in proportion of GHQ-cases was statistically significant according to nationality and parents' occupation (medical professional).

### Sources and self-rated severity of stressors

The most frequently occurring sources of stress were, 'quality of food in mess', 'high parental expectations', 'dissatisfaction with the class lectures', vastness of academic curriculum/syllabus', worrying about the future', 'lack of entertainment in the institution', "frequency of examinations', 'becoming a doctor (expectations on all fronts)', and 'lack of time for recreation'. 'Quality of food in the mess', 'worrying about the future', 'high parental expectations', 'dissatisfaction with the class lectures', 'lack of entertainment' in the institution, 'vastness of academic curriculum/syllabus', and 'frequency of examinations' were rated as most severe. (Table 1)

**Table 1:** Percentage of frequency of occurrence of the 28 sources of stress and perceived severity (rated in a likert scale of 1–10) as reported by the students

**Sources of stress**	**Frequency of occurrence**			**Severity**	
	
	Never/rarely	Sometimes	Often/always	Median	IQR*
Quality of food in mess	17.1	22.5	60.4	8	4–10
High parental expectations	22.4	25.3	52.3	5	3–8
Dissatisfaction with the class lectures	16.9	36.2	46.8	5	4–7
Vastness of Academic curriculum/syllabus	9.3	45.5	45.2	5	4–7
Worry about the future	23.9	31.5	44.6	6	3–8
Lack of entertainment in the institution	29.6	29.4	41	5	3–8
Frequency of examinations	24	36.2	39.8	5	3–7
Becoming a doctor (expectations on all fronts)	26.6	35.3	38.1	5	3–7
Performance in examinations	23.9	40.7	35.4	5	3–7
Lack of time for recreation	27.8	36	36.2	5	3–7
Adjustment with roommate/s	49.2	22.1	28.7	3	1–6
Accommodation away from home	46.7	32.4	25.9	4	2–6
Difficulty in the journey back home	46.8	27.4	25.8	4	2–6
Non-availability of adequate learning materials	40.8	33.4	25.8	4	2–6
Sleeping difficulties (overstrain/disturbances in hostel)	45.3	29.4	25.3	5	2–6
Living conditions in hostel	37.1	38.5	24.4	4	2–7
Lack of special guidance from faculty	36.8	40.7	22.5	4	2–6
Political situation of the country	52.6	25.2	22.2	4	1–6
Competition with peers	56.6	21.7	21.7	5	1–5
Performance in practicals/Clinical postings	51	27	22	3	1–5
Feeling of Loneliness	41.7	38.5	19.8	4	2–6
Relations with the opposite sex	48.9	33.1	18	3	1–5
Financial strain (financial instability in the family)	61.9	23.9	14.1	3	1–5
lllness affecting performances in class and examinations	53.5	34.3	12.3	3	2–5
Difficulty in reading the text books	48.1	39.9	12	4	2–5
Inability to socialize with peers	61.9	27.1	11.1	3	1–5
Family problems (Health related, lack of bonding etc)	74.2	19.5	6.3	2	1–4
Physical disability/limitations	76.4	15.6	8	1	1–4
Alcohol/drug abuse	89.6	7	3.4	1	1–2

* IQR inter quartile range

### Common coping strategies

The five most common coping strategies adopted by the students during the events of stress were 'positive reframing', 'planning', 'acceptance', 'active coping' and 'self distraction'. The descriptive statistics of the common coping strategies adopted by the students are given in Table 2. However, there were significant differences in the coping strategies used by the students according to GHQ-caseness, gender, phase of study (basic or clinical sciences) and occupation of the parents. The students who were GHQ-cases used self blaming, planning, self distraction, denial and venting. Male students used active coping, alcohol/substance use and self blame. Clinical sciences students were using alcohol and instrumental support as coping strategies. Students whose parents were doctors were using planning and emotional support.

**Table 2:** Coping strategies adopted by the students during events of stress

**Coping Mechanism**	**Over all**	**GHQ-cases**	**GHQ-non cases**
	
	**Mean (SD)**	**Mean (SD)**	**Mean (SD)**
Positive reframing	5.85 (1.49)	5.81 (1.44)	5.86 (1.5)
Planning	5.77 (1.55)	6.2 (1.53)	5.65 (1.53)
Acceptance	5.73 (1.62)	5.83 (1.71)	5.7 (1.59)
Active coping	5.68 (1.54)	5.74 (1.34)	5.66 (1.58)
Self distraction	5.06 (1.39)	5.46 (1.43)	4.95 (1.36)
Emotional support	4.80 (1.64)	4.9 (1.82)	4.77 (1.59)
Instrumental support	4.77 (1.68)	4.93 (1.77)	4.72 (1.65)
Religion	4.48 (1.91)	4.6 (2.02)	4.45 (1.87)
Venting	4.34 (1.42)	4.84 (1.55)	4.21 (1.36)
Self blaming	4.23 (1.68)	5.24 (1.81)	3.96 (1.53)
Use of humor	3.78 (1.62)	3.85 (1.76)	3.76 (1.59)
Denial	3.69 (1.59)	4.32 (1.87)	3.53 (1.46)
Behavioral disengagement	3.32 (1.43)	3.73 (1.63)	3.21 (1.36)
Alcohol/drug use	2.50 (1.06)	2.62 (1.22)	2.47 (1.02)

### Predictors of GHQ-caseness

By logistic regression analysis occurrence of academic and health-related stressors were predictors of GHQ-caseness. The odds ratios and 95% confidence intervals of the univariate and multivariate analyses are presented in Table 3. By logistic regression analysis of coping strategies with GHQ-caseness and sources of stress, 'self blame' was associated with GHQ-caseness, venting with academic stressors, 'denial' and 'disengagement' with psychosocial stressors and 'alcohol/drug use' with health-related stressors. (Table 4)

**Table 3:** Predictors of GHQ-caseness by logistic regression analysis

Variable	Number	Number of GHQ Cases (%)	Adjusted OR	(95% CI)
**Gender**				
Male	225	43 (19.1)	1	
Female	173	41 (23.7)	1.31	0.75–2.22
**Phase of study**				
Basic science	239	53 (22.2)	1	
Clinical science	163	31 (19.0)	0.66	0.38–1.14
**Nationality**				
Indian	196	56 (28.6)	1	
Nepali	161	22 (13.7)	0.58	0.31–1.09
Srilankan & others	40	6 (15.0)	0.44	0.17–1.14
**Parents occupation**				
Other professions	285	49 (16.9)	1	
Medical professional	112	35 (31.3)	1.68	0.94–3.01
**Occurrence of academic stressors**				
Less than often	191	27 (14.3)	1	
Often/always	206	57 (27.7)	**2.12**	**1.21–3.7**
**Occurrence of psychosocial stressors**				
Less than often	259	42 (16.2)	1	
Often/always	138	30 (21.7)	1.5	0.89–2.65
**Occurrence of health-related stressors**				
Less than often	317	55 (17.4)	1	
Often/always	80	29 (36.3)	**2.08**	**1.14–3.78**

* information on gender, parents occupation, nationality etc was missing in 6 questionnaires therefore logistic regression analysis was limited to only 397 cases

**Table 4:** Association between stressor groups and coping mechanisms by logistic regression analysis (Only coping mechanism with positive association are shown)

Variable	Mean	SD	Adjusted OR	95% CI
**GHQ-Caseness**				
Self blame	3.78	1.62	1.38	1.15–1.65
**Occurrence of academic stressors**				
Venting	4.34	1.42	1.25	1.04–1.50
**Occurrence of psychosocial stressors**				
Denial	3.69	1.59	1.18	1.01–1.39
Disengagement	3.32	1.43	1.24	1.04–1.47
**Occurrence of health-related stressors**				
Alcohol/drug abuse	2.50	1.06	1.43	1.14–1.80

## Discussion

The psychological morbidity in our study was less compared to studies reported from developed countries [6,9,19,23,24]. In the present study 12-item GHQ was used. Earlier studies from United Kingdom used a more conservative cut off score of 3–4 for allowing comparability of results with other studies from United Kingdom [6-9]. We considered a cut off score of 4–5 to identify probable cases. However, the actual cut off score chosen depends on the purpose and context of each study, and relates to the relative importance of sensitivity and specificity [25]. But some studies on stress have either not used GHQ or used various other instruments for measuring the stress levels among the medical students [12,16,17]. Despite the variability of cut-offs used to estimate the prevalence, psychiatric morbidity reported in our study can be considered as high. The results obtained in our study have clinical importance with regard to the general health status and quality of life of the students. Such a study has not been carried out in the medical schools of Nepal.

A study from Agha Khan University, Pakistan has reported that more than 90% of students felt stressed at one time or the other during their course [12]. A similar study from India reported that 73% of the students had perceived stress at one time or the other during their medical school [16]. Saipanish reported that 61.4% of students in a Thai Medical School had experienced some degree of stress as measured by the Thai Stress Test [17]. Studies from the United Kingdom, Australia and Singapore which have used GHQ have reported different rates of psychological morbidity among medical students [19,23,24]. There are also variations in the sociocultural contexts and the medical curricula of the settings where such studies were carried out. Hence the results of the studies cited above may not be comparable with our findings.

Prevalence of psychological morbidity was higher among Indian students and students whose parents were doctors and this difference was statistically significant. Students from India, Sri Lanka and other countries were living in a new environment and they were also exposed to stressful life conditions in pursuit of a highly demanding professional course especially during the two years of basic science training. In our study there was no significant difference in the prevalence of psychological morbidity according to year to study or phase of study. Among the basic science students, psychological morbidity was higher among the first year students as compared to second year (28.4% against 16.3%). The decrease in prevalence of psychological morbidity in the second year of study can be explained by a gradual adaptation of students to the new living environment and the course.

Among the clinical science students there was an increasing rate among third, fourth and final year students (15%, 18.9% and 24% respectively). Results of our study are similar compared to studies from Pakistan, India and Thailand which have reported a higher level of perceived stress among third and fourth year students [12,16,17]. However the curricula of these medical schools may be different. The prevalence of psychological morbidity among the students whose parents were doctors was higher. We hypothesized that if the parents were medical doctors the students will receive better guidance about the stresses during the course from their parents. On the contrary, the students whose parents were medical doctors had higher prevalence of psychological morbidity. This may probably be due to a high parental expectation which was the second most common source of stress and rated as third in terms of severity.

Occurrence of stressor groups varied with GHQ caseness. Among the students who were GHQ-cases academic, psychosocial and health-related stressor groups occurred more frequently. This suggested that they had a global response to a wide range of potential stressors, rather than to a few specific items. However, by logistic regression analysis psychosocial stressors did not show any association with GHQ-caseness. In terms of frequency of occurrence, academic stressors occurred most frequently, followed by psychosocial and health-related stressors. There were no significant differences in the occurrence of these groups of stressors according to gender, year of study or nationality. However psychosocial stressors occurred more frequently among Indian students. This difference was further evidenced by significant difference in GHQ-caseness according to nationality.

Most students had experienced either academic or psychosocial stressors. Among the academic stressors 'dissatisfaction with the class lectures', 'vastness of academic curriculum/syllabus', 'frequency of examinations' and 'performance in the examinations' occurred more frequently and also the students rated these stressors as severe. Previous studies have also reported that academics/exams are common sources of stress among medical students [6,12-14,16-18,23,26]. Even though 'tests/exams' are the major sources of stress, they are necessary in the medical training as a tool for evaluation/assessment and to encourage student learning. Some students perceive tests/exams as a burden while others consider them helpful for learning.

At MCOMS the present system of evaluation uses subjective questions. The students are marked according to their answers and the results are declared either 'pass' or 'fail' in the examination. In such a system of evaluation students often aim to obtain a 'pass'. This system of evaluation may not measure what a student knows. Sometimes it can be unfair and can damage the student's academic concept and self-esteem. Factors like self-expectation and expectation from their significant 'others' may influence students' perception of their marks. Hence the contents, teaching and learning methods, and the evaluation process, needs to be analyzed and improved. The teaching-learning schedule of medical students should be modified to encourage more student participation.

In our study psychosocial factors also played an important role in psychological morbidity. The important psychosocial factors were 'quality of food in mess', 'high parental expectations', 'lack of entertainment', 'feeling of loneliness and 'worrying about the future' and these factors may be linked to staying in the hostel. Majority of our students were residing in the hostels provided in the campus. Earlier studies have reported that psychosocial factors are important sources of stress for medical students [16,17,19]. There may be a need to provide more time and facilities in the campus for recreation and sports. Although these facilities were available in our institution they were felt to be inadequate by the students. These factors should be explored in detail in a future study. A longitudinal qualitative study among Swedish medical school has reported that stress and burn out is determined by individual traits and school environment [27]. The study also recommended that individual and organizational interventions may be for prevention of burn out among medical students. A study from US medical school reported that an elective in 'Mind-Body Medicine may decrease anxiety scores among preclinical medical students [28]. Another study from US has recommended that teaching stress management and self-care skills to medical students may be successful [29].

Coping strategies refer to the specific efforts, both behavioral and psychological, that people employ to master, reduce tolerate or minimize stressful events. 'Active coping' means taking action or exerting efforts to remove or circumvent the stressor, 'acceptance' means accepting the fact that the stressful event had occurred and is real while 'planning' consists of thinking about how to confront the stressor and planning one's coping efforts 'positive reframing' means making the best of the situation by growing from it or seeing it in a more positive light, 'denial' is an attempt to reject the reality of the stressful event while 'behavioral disengagement' means giving up or withdrawing efforts from the attempt to attain the goal with which the stressor is interfering [22]. The students in our study adopted active coping strategies (positive reframing, planning, acceptance, and active coping) rather than avoidant strategies (denial, alcohol/drug use and behavioral disengagement). Studies from the United Kingdom have reported, use of alcohol, tobacco and drugs as common coping strategies adopted by the medical students [6-11]. It is encouraging to note that in our study alcohol/drug use was the least common coping strategy. However, we could not rule out under reporting of such behaviour by students in spite of assurance of anonymity and confidentiality of their responses by the investigators involved in the present study.

The students who were GHQ-cases used a mix of both active and avoidant coping strategies but male students were using active coping and alcohol/drug more often than females. The students tended to use alcohol/drugs more often in the clinical years than in the basic sciences when they were more disciplined. During the clinical science years the students used instrumental support as coping strategy. It is possible that during the clinical science years they are well acquainted with their seniors and take their advice for solving their academic and day-to-day problems.

The mental health status of the students was assessed during previous time period of a few weeks only. We carried out this survey during the middle of the semester to avoid the stressful time of sessional and university examinations at the end of semester. However in our medical school assessment/evaluation is ongoing with frequent evaluation of student learning. Therefore, the stress status measured may represent the natural level of stress among medical students.

Cross-sectional design did not allow us to study the cause-and-effect relationship of psychological morbidity with stress and coping strategies. Therefore a prospective study is necessary to study such relation. Despite good response rate, another limitation of our study may be that of non-response bias. It would have been advantageous to interview a sample of non-respondents to assess their experiences and psychological status. Anonymity and confidentiality of the respondents was ensured in the questionnaire. Hence we did not have the identity of these students to interview the non-responders on a separate occasion. The various stressors were only listed and their details and possible amelioration strategies were not explored.

## Conclusion

A large proportion of students in both clinical and basic science have potential psychological problems. The stressors experienced by the students were mainly related to academics and psychosocial concerns. These stressors need to be analyzed further. There is need to address these stressors by student advisors, peer education and counseling. The coping strategies commonly used by the students were positive reframing, planning, acceptance, active coping self distraction and emotional support. No new coping strategies were discovered in our Nepalese setting. The students should be taught different stress management techniques to improve their ability to cope with a demanding professional course. The living conditions of the students and their recreational facilities should be improved. There is also need to bring about changes in the quality of teaching and evaluation system.

## Competing interests

The author(s) declare that they have no competing interests.

## Authors' contributions

CTS conceived designed and carried out the study and prepared the first draft of the manuscript. PR helped in designing the study carried out the research and helped in writing the manuscript. BVS helped in carrying out the research, performed data analysis, interpretation of the results and helped in drafting the manuscript. CM designed the Questionnaire, helped to conceive the research, and prepared the paper for submission. BR helped in conceiving the research, designing the Questionnaire, prepared the paper for submission. RGM administered the questionnaire, assisted in data entry and analysis, and criticized the earlier drafts of the manuscript.

## Pre-publication history

The pre-publication history for this paper can be accessed here:



## References

[B1] Fish C, Nies MA (1996). Health promotion needs of students in a college environment. Public Health Nurs.

[B2] Chew-Graham CA, Rogers A, Yassin N (2003). 'I wouldn't want it on my CV or their records': medical students' experiences of help-seeking for mental health problems. Med Educ.

[B3] Silver HK, Glicken AD (1990). Medical student abuse. Incidence, severity, and significance. JAMA.

[B4] Niemi PM, Vainiomaki PT (1999). Medical students' academic distress, coping and achievement strategies during the pre-clinical years. Teach Learn Med.

[B5] Vitaliano PP, Russo J, Carr JE, Heerwagen JH (1984). Medical school pressures and their relationship to anxiety. J Nerv Ment Dis.

[B6] Guthrie EA, Black D, Shaw CM, Hamilton J, Creed FH, Tomenson B (1995). Embarking upon a medical career: psychological morbidity in first year medical students. Med Educ.

[B7] Firth J (1986). Levels and sources of stress in medical students. BMJ.

[B8] Guthrie EA, Black D, Shaw CM, Hamilton J, Creed FH, Tomenson B (1997). Psychological stress in medical students: a comparison of 2 very different university courses. Stress Med.

[B9] Guthrie E, Black D, Bagalkote H, Shaw C, Campbell M, Creed F (1998). Psychological stress and burnout in medical students: a 5-year prospective longitudinal study. J Roy Soc Med.

[B10] Miller P, Surtees PG (1991). Psychological symptoms and their course in first year medical students as assessed by the Interval General Health Questionnaire (I-GHQ). Br J Psychiatry.

[B11] Ashton CH, Kamali F (1995). Personality, lifestyles, alcohol and drug consumption in a sample of British medical students. Med Educ.

[B12] Shaikh BT, Kahloon A, Kazmi M, Khalid H, Nawaz K, Khan N, Khan S (2004). Students, stress and coping strategies: a case of Pakistani medical school. Educ Health (Abingdon).

[B13] Stewart SM, Betson C, Marshall I, Wong CM, Lee PWH, Lam TH (1995). Stress and vulnerability in medical students. Med Educ.

[B14] Stewart SM, Lam TH, Betson CL, Wong CM, Wong AMP (1999). A prospective analysis of stress and academic performance in the first 2 years of medical school. Med Educ.

[B15] Stewart SM, Betson C, Lam TH, Marshall IB, Lee PWH, Wong CM (1997). Predicting stress in first year medical students: a longitudinal study. Med Educ.

[B16] Supe AN (1998). A study of stress in medical students at Seth G.S. Medical College. J Postgrad Med.

[B17] Saipanish R (2003). Stress among medical students in a Thai medical school. Med Teach.

[B18] Sherina MS, Rampal L, Kaneson N (2004). Psychological stress among undergraduate medical students. Med J Malaysia.

[B19] Moffat KJ, McConnachie A, Ross S, Morrison JM (2004). First year medical student stress and coping in a problem-based learning medical curriculum. Med Educ.

[B20] Kathmandu University (2001). Curriculum for MBBS part one. Basic medical sciences.

[B21] Goldberg DP (1978). Manual of the General Health Questionnaire.

[B22] Carver CS (1997). You want to measure coping but your protocol's too long: consider the brief COPE. Int J Behav Med.

[B23] Ko SM, Kua EH, Fones CS (1999). Stress and the undergraduates. Singapore Med J.

[B24] Willcock SM, Daly MG, Tennant CC, Allard BJ (2004). Burnout and psychiatric morbidity in new medical graduates. Med J Aust.

[B25] Wilkin D, Hallam L, Doggett M (1994). Measures of need and outcome for primary health care.

[B26] Coles C (1994). Medicine and stress. Med Educ.

[B27] Dahlin ME, Runeson B (2007). Burnout and psychiatric morbidity among medical students entering clinical training: a three year prospective questionnaire and interview-based study. BMC Med Educ.

[B28] Finkelstein C, Brownstein A, Scott C, Lan YL (2007). Anxiety and stress reduction in medical education: an intervention. Med Educ.

[B29] Redwood SK, Pollak MH (2007). Student-led stress management program for first-year medical students. Teach Learn Med.

